# Clinical Relevance of HLA Antibodies in Kidney Transplantation: Recent Data from the Heidelberg Transplant Center and the Collaborative Transplant Study

**DOI:** 10.1155/2017/5619402

**Published:** 2017-06-04

**Authors:** Caner Süsal, Alexander Fichtner, Burkhard Tönshoff, Arianeb Mehrabi, Martin Zeier, Christian Morath

**Affiliations:** ^1^Transplantation Immunology, Institute of Immunology, University of Heidelberg, Heidelberg, Germany; ^2^Department of Pediatrics I, University Children's Hospital, University of Heidelberg, Heidelberg, Germany; ^3^Department of Transplantation and General Surgery, University of Heidelberg, Heidelberg, Germany; ^4^Division of Nephrology, University of Heidelberg, Heidelberg, Germany

## Abstract

Herein, we summarize our recent findings from the international Collaborative Transplant Study (CTS) and Heidelberg Transplant Center regarding the role of HLA antibodies in kidney transplantation and their application into the clinical routine. Based on the antibody findings from the CTS serum study, an algorithm was developed in 2006 for the transplantation of high-risk sensitized patients at the Heidelberg Transplant Center which includes seven different pre- and posttransplant measures. Using this algorithm, the number of transplantations could be increased in high-risk presensitized patients and the previously existing impact of antibodies on graft survival could greatly be diminished but not totally eliminated. More recent findings led to the hypothesis that T cell help from a preactivated immune system supports the harmful effects of pretransplant donor-specific HLA antibodies that otherwise disappear in many cases after transplantation without any consequence.

## 1. Introduction

Although effective therapies to treat antibody-mediated rejection (AMR) still need to be developed, with his vision and strong determination, Paul Ichiro Terasaki was the driving force that convinced the transplant community to perform the necessary studies to comprehend the different aspects of humoral rejection in kidney transplantation. We dedicate this article therefore to this great scientist.

Thanks to the introduction of the single-antigen bead technique (SAB), which allows detection of HLA antibodies with high sensitivity, and improvement of pathological diagnosis, we widely understand today the role of donor-specific HLA antibodies (DSA) in the posttransplant phase. However, in which patients pretransplant DSA would exert their harmful effects is still not fully understood. Many patients were transplanted in the past in the presence of preexisting DSA; not all of them lost their grafts, even if the DSA was strong and complement-activating [1–3]. Pretransplant DSA disappear in many patients without any clinical consequence directly after transplantation, whereas in others, even weak pretransplant DSA persist and do harm in the subsequent course [3, 4].

## 2. Presensitization as a Major Problem

Kidney transplantation of presensitized patients with HLA antibodies in their serum is challenging mainly for two reasons. (1) To prevent a positive preoperative complement-dependent cytotoxicity (CDC) crossmatch result and diminish the harmful effects of pretransplant DSA, unacceptable HLA antigen mismatches are determined using sensitive assays and in the consequence many organ offers are excluded for these patients already at the virtual crossmatch level. Without further measures, presensitized patients accumulate on the kidney waiting list and face prolonged waiting times. (2) Even when the pretransplant CDC crossmatch result is negative and the patient is successfully transplanted, long-term graft survival may be impaired in these patients, due to either persistence or reappearance of pretransplant DSA in the posttransplant phase or development of de novo DSA which can cause antibody-mediated tissue injury.

## 3. Heidelberg Algorithm for Transplantation of Presensitized High-Risk Patients

**Figure 1:**
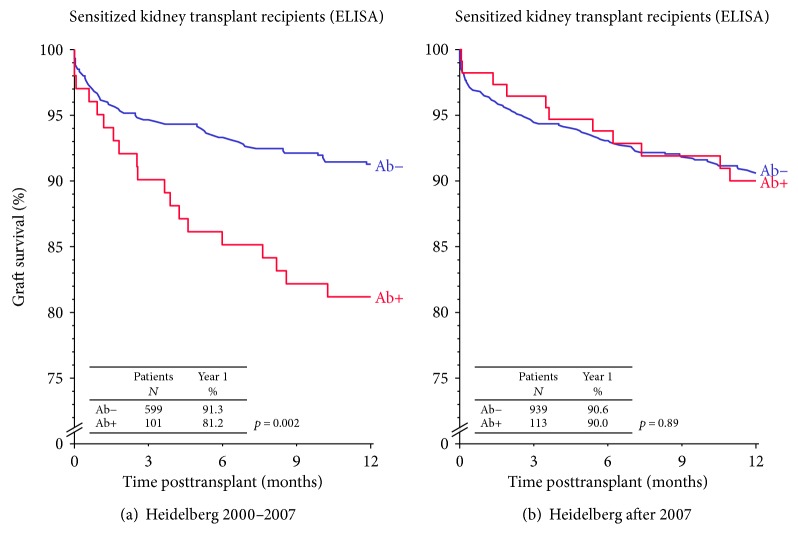
Graft survival in patients with and without ELISA-reactive HLA antibodies who were transplanted at the Heidelberg Transplant Center between 2000 and 2007 (a) and after 2007 (b). Ab: ELISA-reactive HLA antibody.

To overcome these two major problems, we introduced in April 2006 an algorithm for the transplantation of presensitized high-risk kidney transplant recipients at our center and adapted it further in 2007, 2009, and 2016 [3, 5–7]. A total of seven different measures are used in an integrated fashion to transplant these patients in a reasonable period of time with improved outcomes (Table 1). As shown in Figure 1(a), presensitized patients with ELISA-reactive HLA antibodies who were transplanted in the years 2000 to 2007 showed significantly lower graft survival rates than patients without ELISA-reactive HLA antibodies. This difference disappeared after the introduction of the Heidelberg Algorithm although more high-risk patients were transplanted (Figure 1(b)).

The most critical components of our integrative approach are the pretransplant identification of high-risk patients on the waiting list (measure 1) and the risk-stratified organ allocation (measures 2 and 3). For example, a patient who has a high cytotoxic PRA and/or is positive for both class I and II HLA antibodies in ELISA (measure 1) is at increased risk of graft loss. We reported in two independent series of 4136 and 5315 kidney transplant recipients on the increased risk of graft loss in the presence of pretransplant class I and class II HLA antibodies, as measured by ELISA [8, 9]. These patients may be successfully and timely transplanted when only a low number of HLA mismatches are present (measure 2) [9], and the transplantation is performed via the Eurotransplant Acceptable Mismatch program which allocates organs to highly immunized patients with high priority (measure 3) [10]. Since October 2016, pretransplant determination of the immune activation marker soluble CD30 (sCD30) in ELISA has also become an important component of pretransplant risk estimation in measure 1 of the Heidelberg Algorithm because pretransplant activation of the immune system, as measured by high sCD30 levels, was in two recent studies of 80 presensitized high-risk patients from Heidelberg and 385 presensitized patients from 13 transplant centers (corresponding to a series of some 1000 patients) found to be a substantial risk factor for graft loss in the presence of DSA (see below) [3, 7].

All as “high-risk”-categorized patients receive, during a deceased-donor organ offer process or in preparation for transplantation from a living donor, pre- and postoperative apheresis treatment (measures 4 and 5) to bring undetected antibody to a lower level and to prevent antibody-mediated allograft injury due to an early rebound of preexisting DSA. To prevent the development of de novo DSA, this is combined with the administration of anti-B cell antibody rituximab (measure 4). B cells are important antigen-presenting cells and are critical for T cell activation and the development of T cell memory during alloimmune responses. Despite a lack of effect against long-lived plasma cells, in some reports, anti-CD20 therapy was associated with a reduction of DSA reactivity. Rituximab may prevent the generation of antibody-producing cells from the naive B cell pool and may target short-lived plasma cells that express CD20 on their surface. In addition, anti-CD20 therapy may deplete B cell aggregates within allografts. Kohei et al. reported on 1.7% and 18.1% de novo DSA rates in patients, after ABO-incompatible or ABO-compatible kidney transplantation, indicating that targeting B cell immunity at the time of transplantation with rituximab may reduce antibody-mediated allograft injury during the subsequent course [11]. In our first series of 34 high-risk patients, severe cellular rejection was infrequent under the usage of rituximab, even in the absence of thymoglobulin, while borderline changes were found frequently [5]. Since 2009, high-risk patients in addition receive T cell-eliminating induction therapy by thymoglobulin to target an early T cell response which would support de novo DSA and C1q-DSA development (see below). Protocol biopsies at days 7 and 90 (measure 6) and posttransplant antibody monitoring (measure 7) to diagnose AMR after successful kidney transplantation in its earlier stages complete the Heidelberg Algorithm.

Posttransplant antibody monitoring has recently been further refined with the introduction of the C1q assay. Patients with high mean fluorescence intensity (MFI) DSA of greater than 3000 are automatically tested since March 2016 for the presence of complement C1q component-binding DSA. We also consider posttransplant appearance of C1q-DSA a major risk factor for AMR-mediated graft loss during the further course (see below) [2–4].

Using this approach, even high-risk sensitized patients can be transplanted with graft survival rates that are not different from those of nonsensitized kidney recipients. In our initial analysis, 1-year graft survival, death-censored graft survival, and patient survival rates in 28 deceased donor kidney recipients were 92%, 96%, and 96%, respectively, and no graft loss or patient death was observed in the 6 living-donor kidney recipients [5]. AMR occurred in 1 living and 2 deceased donor kidney transplant recipients during the follow-up. However, the rate of cellular rejections (including borderline changes) in kidney graft biopsies and delayed graft function (DGF) were with 79% and 41%, respectively, quite high. We had previously reported that besides increased cold ischemia time, HLA antibodies and mild forms of AMR may also be involved in DGF [12]. To reduce this high rate of cellular rejection that may initiate AMR and DGF, interleukin-2 receptor antibody induction therapy was substituted by more potent thymoglobulin induction in high-risk sensitized patients from April 2009. This therapy is accompanied by rigorous infection prophylaxis by valganciclovir (when the donor is CMV-positive) and cotrimoxazole (in all patients).

## 4. Association of Posttransplant DSA with Graft Loss

We addressed the clinical value of posttransplant DSA monitoring which is the seventh measure of the Heidelberg Algorithm in three different cohorts: (1) in the CTS serum study, (2) in the Heidelberg pediatric cohort, and (3) in the Heidelberg presensitized high-risk population that was transplanted using the Heidelberg Algorithm.

### 4.1. CTS Data on the Impact of Posttransplant DSA

In the CTS serum study, we investigated a possible association of de novo development and persistence or loss of preexisting DSA with graft failure in 83 patients with failed kidney transplants and in 83 control patients without graft loss who were matched for eight different parameters, including the time after transplantation [4]. We chose this study design, because DSA determinations are costly and graft loss has increasingly become a rare event, and we wanted to include as many patients with graft loss into the analysis as possible. Eighty-three patients with graft loss correspond to a series of some 1000 transplant recipients. Antibody reactivity at five different cutoffs (500, 1000, 2000, 3000, and 5000 MFI) was evaluated systematically, and available recipient and donor DNA allowed the precise determination of DSA against 10 different HLA loci.

In this study, the rate of de novo DSA and also non-DSA with ≥500 MFI was higher in the graft loss than in the nonrejector group (76% versus 40%, *p* < 0.001). Because of the low number of patients developing de novo DSA (22% of patients with graft loss), the DSA results did not reach statistical significance. At all cutoffs, there was a significantly higher rate of de novo non-DSA in patients with graft loss, which was explained rather by adsorption of DSA onto the graft than epitope sharing. Furthermore, the incidence of strong pretransplant DSA with 5000 MFI or higher that persist after transplantation was also higher in the graft loss group (10% versus 1%, *p* = 0.034).

The main problem in the clinical routine is that de novo DSA appear also in patients without immediate graft loss. When the C1q-binding ability of de novo or persistent DSA was analyzed in sera of patients with and without graft loss, none of the nonrejectors demonstrated C1q positivity, whereas 43% of patients with graft loss showed C1q-positive antibodies, although not necessarily donor-specific (*p* < 0.001). Overall, our data from this study indicated that the posttransplant presence of persisting or de novo HLA antibodies, especially if strong and C1q-binding, is associated with graft loss, even if the antibodies are not specific for mismatched donor HLA [4].

### 4.2. Evaluation of Posttransplant DSA Monitoring in Pediatric Patients with Indication Biopsy

Antibody effects appear to be stronger in pediatric than adult recipients [13]. Therefore, we found it important to investigate also in our pediatric cohort the diagnostic value of posttransplant DSA.

**Figure 2:**
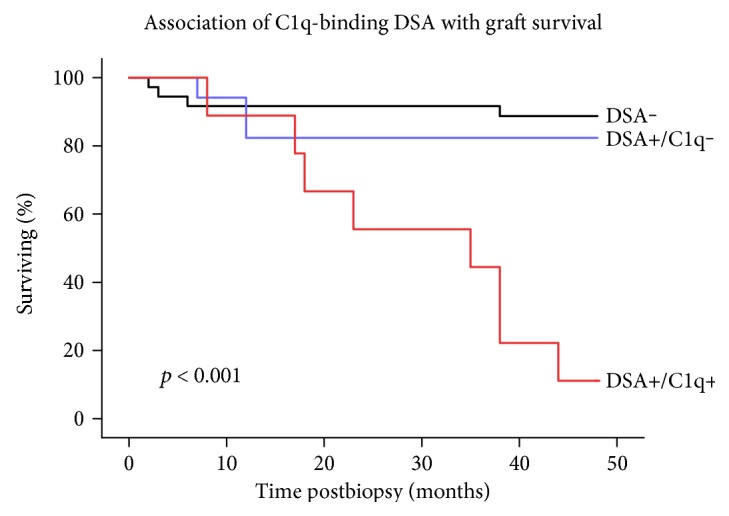
Postbiopsy kidney graft survival in pediatric patients stratified according to the donor-specific HLA antibody- (DSA-) C1q status at the time of indication biopsy. Patients with C1q-DSA positivity had a significantly inferior graft survival compared to patients without DSA (*p* < 0.001). Patients with DSA but without C1q positivity showed comparable graft survival to DSA-negative patients (*p* = 0.55) but significantly better graft survival than patients with C1q-DSA positivity (*p* = 0.001). Modified from [14].

Sera of 62 patients who underwent clinically indicated graft biopsies were tested for DSA, and their association with specific histological lesions and subsequent graft outcome was analyzed [14]. Twenty-six patients (42%) were DSA-positive at the time of indication biopsy and nine (15%) of them were in addition C1q-positive. At 4 years after biopsy, the nine patients with C1q positivity showed a graft survival rate of 11%, which was strikingly lower than the 88% and 82% survival rates in DSA-negative and DSA-positive but C1q-negative patients, respectively (*p* < 0.001 and *p* = 0.001, resp.) (Figure 2). The majority (89%) of C1q-positive patients in this study were diagnosed with chronic active AMR. C1q-positive DSA (adjusted hazard ratio (HR) = 6.4), presence of transplant glomerulopathy (HR = 9.5), and estimated glomerular filtration rate (eGFR) at the time of indication biopsy (HR = 0.9) were risk factors for subsequent graft loss. Thus, the presence of C1q-positive DSA in the context of an indication biopsy identified a subgroup of pediatric renal transplant recipients with a markedly increased risk of subsequent graft loss. Because a fraction of DSA-positive patients escape rejection or graft dysfunction, the C1q assay appeared to increase the specificity of a positive DSA result regarding unfavorable transplant outcome.

### 4.3. Impact of Posttransplant DSA in the Heidelberg High-Risk Collective

Our adult high-risk cohort which consists of patients who are transplanted via the Heidelberg Algorithm is a special population in which the antibody effects are expected to occur in an accelerated manner. Compared to that of the international CTS study, we have in this cohort low number of patients with graft loss but more precise information on individual patients. Recently, we analyzed 80 of these high-risk sensitized patients who were transplanted at our center from April 2006 to November 2011 with a minimum follow-up for all patients of 36 months [3].

**Figure 3:**
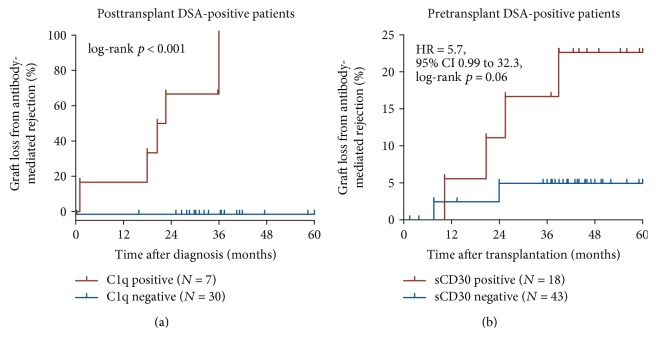
Graft loss from antibody-mediated rejection in high-risk sensitized patients with and without C1q-binding posttransplant donor-specific HLA antibodies (DSA) (a) and in patients who in addition to pretransplant DSA positivity had also increased levels of the immune activation marker sCD30 before transplantation (b).

Despite all measures, seven patients developed AMR and six of them lost their graft within the first 4 years after transplantation, and all six patients were positive for C1q-DSA (1 persistent, 4 de novo, and 1 persistent plus de novo C1q-DSA) (Figure 3(a)).

In contrast to this striking association between posttransplant C1q-DSA and AMR-related graft loss, the predictive value of pretransplant C1q-DSA was, even in this high-risk group, quite low. Of the 61 patients with pretransplant DSA (cutoff 500 MFI), 14 patients possessed C1q-DSA (cutoff 300 MFI). AMR rates and AMR-related graft loss in patients with pretransplant C1q-DSA were with 36% versus 28% and 14% versus 8%, respectively, not significantly different from the rates in patients with C1q-negative DSA. Interestingly, as many as 11 of 13 (85%) high-risk patients with pretransplant C1q-DSA and a posttransplant serum lost their C1q-DSA after surgery with an unremarkable clinical course, which is in line with the findings of Otten et al. and Loupy et al. that posttransplant but not pretransplant C1q-DSA predict AMR and AMR-related graft loss [1, 2].

## 5. Pretransplant DSA and sCD30

Earlier data from our group and others indicated that a preactivated immune system, as measured by sCD30, especially in combination with HLA antibodies is a good indicator of posttransplant rejection and graft loss [15–19]. Allostimulation results in the upregulation of the T cell activation marker CD30 on CD4 as well as CD8 memory T cells and increased release of the 88 kD sCD30 from these cells in an IFN-*γ*- and IL-2-dependent manner [20]. In the search for further biomarkers to improve risk estimation before transplantation as the basic component of Heidelberg Algorithm, we recently investigated a possible association of sCD30, DSA, and antibody-mediated graft loss in the group of 80 high-risk sensitized patients. The risk for AMR-related graft loss in 18 patients who had both, a positive pretransplant DSA value (cutoff 500 MFI) and a positive sCD30 value (cutoff 100 ng/mL), was 11 times higher than that in the remaining 62 patients (HR = 11.1, 95% CI 1.68 to 73.4, log-rank *P* = 0.013) and 5.7 times higher than that in DSA-positive but sCD30-negative patients (Figure 3(b)). Two patients who were sCD30-negative pretransplant and experienced AMR-related graft loss had a gap in immunosuppressive therapy and became sCD30-positive (posttransplant cutoff 40 ng/mL) prior to their AMR episode [3].

**Figure 4:**
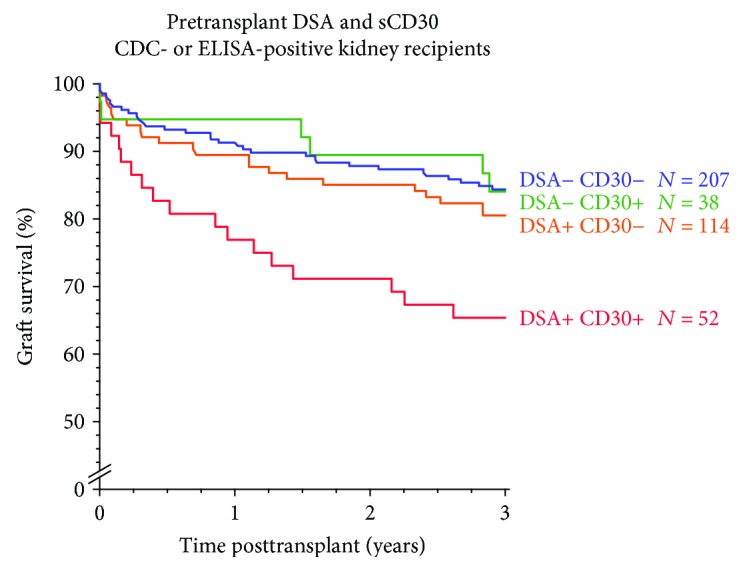
Impact of pretransplant sCD30 on graft survival in patients with and without pretransplant donor-specific HLA antibodies (DSA) in single-antigen bead testing. An update of the results from [7] is shown.

To substantiate this finding, we analyzed the combined impact of pretransplant DSA and the immune activation marker sCD30 on a larger cohort of 385 presensitized kidney transplant recipients from the CTS database who possessed ELISA- or CDC-reactive HLA antibodies in their serum [7]. In this study, a deleterious influence of pretransplant DSA (cutoff 1000 MFI) on 3-year graft survival was evident only in patients who were positive (≥80 ng/mL) for the immune activation marker sCD30. In the absence of sCD30 positivity, 3-year graft survival was almost identical in patients with or without DSA (83% and 84%, *P* = 0.81). In contrast, a strikingly lower 3-year graft survival rate of 62% was observed in patients who were both sCD30- and DSA-positive (HR 2.9, *P* < 0.001). Even in the presence of strong DSA with ≥5000 MFI, the 3-year graft survival rate was high if the recipients were sCD30-negative. An update of these results in 411 patients is shown in Figure 4.

However, our findings on the clinical relevance of SAB-detected pretransplant DSA and sCD30 are restricted to presensitized patients with CDC- or ELISA-reactive antibodies. We reported previously on the missing association of SAB-detected pretransplant DSA with graft loss in CDC- and ELISA-negative kidney graft recipients [21], which could partly be explained by false positive results due to reactivity with denatured antigen on the beads that can be observed in healthy individuals [22] as well as in kidney transplant recipients without history of an immunizing event [23].

We hypothesize that patients with pretransplant DSA and the activated immune system (as measured by pretransplant sCD30) require special attention after kidney transplantation. In these patients, a gap in immunosuppressive therapy may lead to persistence, reappearence, or de novo occurrence of strong, complement-activating DSA, resulting in severe AMR and, without immediate intervention, in AMR-related graft loss.

## 6. Conclusions

Integrated approaches are required for successful and timely transplantation of presensitized high-risk patients. Estimation of risk of graft failure prior to transplantation is important and requires further precision by introduction of additional biomarkers. Combination of DSA in presensitized patients with CDC- or ELISA-reactive antibodies with the immune activation marker sCD30 appears promising and deserves further evaluation.

## Figures and Tables

**Table 1:** “Heidelberg Algorithm” (applied since April 2006).

(1) Pretransplant identification of high-risk patients
*Donor-independent*
(i) CDC-PRA-DTT ≥85% (current or historical) (ii) HLA class I and II antibody positivity in ELISA (iii) HLA class I positivity in ELISA (retransplant)
*Donor-dependent*
(i) Positive CDC B-cell crossmatch in retransplant recipients with HLA class II antibody positivity in ELISA (ii) Positive CDC T-cell crossmatch (iii) DSA ≥1,000 MFI (living donor; since April 2009) (iv) DSA ≥1,000 MFI and sCD30 ≥ 80 ng/ml (since October 2016)

(2) Good HLA match in patients with HLA class I and class II antibody positivity in ELISA (deceased donor)
(i) CDC-PRA-DTT ≥10%: 0-1 HLA-A, -B, -DR mismatches (ii) CDC-PRA-DTT <10%: 0-2 HLA-A, -B, -DR mismatches

(3) Acceptable Mismatch Program of Eurotransplant (deceased donor)
(i) CDC-PRA-DTT ≥85% (current or historical)

(4) Pretransplant treatment
(i) Single plasmapheresis (deceased donor) (ii) Repeated immunoadsorption (living donor) (iii) Triple immunosuppression (tacrolimus + enteric-coated mycophenolic sodium + methylprednisolone; in the case of living donor, together with the initiation of apheresis therapy) (iv) Rituximab 375 mg/m^2^ (when all crossmatches are negative) (v) Thymoglobulin 1.5 mg/kg body weight preoperatively and a median of 2 times (range: 1–6) postoperatively (since April 2009; IL-2 receptor antagonist basiliximab before April 2009)

(5) Posttransplant treatment
(i) Repeated plasmapheresis (deceased donor) (ii) Repeated immunoadsorption (living donor)

(6) Protocol biopsies
(i) On days 7 and 90 (since November 2007)

(7) Posttransplant monitoring of DSA
(i) On days 0, 7, 30, 180, and every 6 months thereafter (ii) If deterioration of allograft function (iii) C1q assay if DSA ≥3,000 MFI (since March 2016)

Adopted from [5]. CDC: complement-dependent cytotoxicity; PRA: panel reactive antibodies; DTT: dithiothreitol; DSA: donor-specific HLA antibodies; sCD30: soluble CD30.
